# Effects of Raloxifene on Bone Metabolism in Hemodialysis Patients

**DOI:** 10.5812/ijem.5367

**Published:** 2012-06-30

**Authors:** Jun Iwamoto

**Affiliations:** 1 Institute for Integrated Sports Medicine, Keio University School of Medicine, Tokyo, Japan

**Keywords:** Raloxifene, Alkaline Phosphatase, Quantitative Ultrasound, Speed of Sound, Chronic Kidney Disease

Dear Editor,

Saito et al. (1) reported that raloxifene (RAL) suppressed bone turnover and increased the quantitative ultrasound (QUS) parameter, speed of sound (SOS) of the calcaneus in hemodialysis postmenopausal women with type 2 diabetes or without type 2 diabetes. Baseline bone turnover markers like serum cross-linked N-terminal telopeptides of type I collagen (NTX) and bone-specific alkaline phosphatase (BAP) indicated high bone turnover in hemodialysis postmenopausal women, and the response of these bone turnover markers to RAL treatment did not differ significantly between diabetic and non-diabetic patients. No adverse events were detected in any patients. Thus, RAL was suggested to be beneficial in improving bone turnover and bone mass in hemodialysis postmenopausal women regardless of the presence of type 2 diabetes. However, owing to the small sample size and short duration of observation (one year), the incidence of fragility fractures was not assessed.

Hemodialysis patients (chronic kidney disease [CKD] stage 5) have renal osteodystrophy, reflecting increased bone turnover caused by secondary hyperparathyroidism. Bone turnover is further increased in hemodialysis postmenopausal women compared with hemodialysis men because of the menopause-related increase in bone turnover. Although bone formation may be decreased in diabetic patients as indicated by low serum insulin-like growth factor-I (IGF-I) and osteocalcin concentrations (2-4), hemodialysis postmenopausal women showed high bone turnover similarly in patients with or without type 2 diabetes in the study conducted by Saito et al. (1) so that the effect of RAL on bone turnover markers was similar in diabetic and non-diabetic patients.

Osteoporosis most commonly affects postmenopausal women, placing them at a significant risk of fractures. RAL is widely used for the treatment of postmenopausal osteoporosis. The Multiple Outcomes of Raloxifene Evaluation (MORE) study, a double-blind, placebo-controlled randomized clinical trial encompassing 7705 postmenopausal women (aged 31–80 years) with osteoporosis, has shown that RAL increases bone mineral density (BMD) at the spine and femoral neck and reduces the risk of vertebral fracture in women with postmenopausal osteoporosis (5). Short stature, age, years since menopause, impaired cognitive function, visuospatial capabilities, impaired musculoskeletal strength, low femoral neck BMD, and prior vertebral fracture are significant factors affecting the incidence of new vertebral fractures (6). Recently, however, researchers have paid attention to CKD and type 2 diabetes in postmenopausal osteoporotic women treated with RAL, because establishing a strategy for the prevention of fragility fractures in postmenopausal osteoporitic women with CKD or type 2 diabetes has been suggested to be a matter of great urgency.

An increased burden of osteoporosis exists in elderly people with CKD. CKD is associated with an increased risk of fragility fractures (7-11). CKD may lead to metabolic abnormalities that accelerate bone loss, such as chronic metabolic acidosis, hypogonadism, hyperparathyroidism, and abnormalities in vitamin D metabolism (12). Thus, the risk of fractures is considered to be higher in postmenopausal osteoporotic women with CKD than in those without CKD.

Treatment approved for osteoporosis in postmenopausal women may be used in patients with stage 1–3 CKD (13). Consensus on the diagnosis of osteoporosis among those with advanced CKD stage 5 patients on dialysis is lacking (13). Because treatment decisions are difficult in stage 5 CKD, bone biopsy may be required and most treatments would be off-label (13). However, the results of the study conducted by Saito et al. (1) suggest the beneficial effect of RAL on bone turnover in hemodialysis postmenopausal women who show high bone turnover.

According to the post-hoc analyses of the MORE study, RAL increases BMD at both the hip and the spine and reduces the risk of vertebral fracture among individuals with CKD (14). The effect of RAL on hip BMD is greater among those with mild to moderate CKD (14). RAL slows a yearly rate of increase in creatinine and a yearly rate of decrease in estimated glomerular filtration rate (eGFR) (15). These findings suggest the efficacy, safety and renoprotective effect of RAL.

The number of patients with type 2 diabetes is increasing. Type 2 diabetes is associated with significantly increased risks of non-vertebral (relative risk: 1.2), hip (relative risk: 1.7), and foot (relative risk: 1.3) fractures (16). Patients with type 2 diabetes are susceptible to low-trauma fractures, especially hip fractures, despite having a normal or high BMD (17). The existence of an increased risk of fractures in type 2 diabetes suggests the potential involvement of other pathogenic influences on bone (e.g., hyperglycaemia, diabetic complications and lifestyle factors) than BMD (17). Because bone strength primarily reflects the integration of BMD and bone quality, the increased risk of fractures in type 2 diabetic patients may be partly explained by poor bone quality. The risk of fractures is considered to be higher in postmenopausal osteoporotic women with type 2 diabetes than in those without type 2 diabetes.

Another analysis of the MORE study has shown that the risk reduction of vertebral fracture after RAL treatment is greater in patients with type 2 diabetes (odds ratio: 0.13, 95 % confidence interval: 0.03–0.55) than in those without type 2 diabetes (odds ratio: 0.49, 95 % confidence interval: 0.49–0.71), suggesting a higher efficacy of RAL against vertebral fractures in patients with type 2 diabetes (6). A preclinical study using ovariectomized rabbits showed that RAL ameliorated hyperhomocysteinemia-induced detrimental cross-linking and improved bone strength via the amelioration of collagen cross-links (18). Collagen cross-link formation is recognized as one of the factors that determine bone quality. Impaired enzymatic crosslinking and/or an increase in non-enzymatic cross-links (advanced glycation end-products [AGEs]) such as glucosepane and pentosidine in bone collagens is proposed to be a determinant of impaired bone mechanical properties in aging, osteoporosis, and diabetes (18). The efficacy of RAL against vertebral fractures in patients with type 2 diabetes may partly be attributable to its effect on bone quality including the collagen-cross linking profile.

The endpoint of treatment for osteoporosis is to prevent fractures. Saito et al. (1) clearly showed the effect of RAL on surrogate markers. I would like to encourage them to assess the prevalence and incidence of vertebral fracture, homocystein, pentosidine (one of the AGEs), renal function, and BMD measured by dual energy x-ray absorptiometry in hemodialysis postmenopausal women treated with RAL.
